# AllEnricher: a comprehensive gene set function enrichment tool for both model and non-model species

**DOI:** 10.1186/s12859-020-3408-y

**Published:** 2020-03-17

**Authors:** Du Zhang, Qi Hu, Xinxing Liu, Kai Zou, Emmanuel Konadu Sarkodie, Xueduan Liu, Fei Gao

**Affiliations:** 1 School of Minerals Processing and Bioengineering, Central South University, Changsha, 410083 China; 2 NEOMICS Institute, Shenzhen, 518122 China; 3 Comparative Pediatrics and Nutrition, Department of Veterinary and Animal Sciences, University of Copenhagen, DK-1870 Frederiksberg C, Denmark

**Keywords:** Enrichment analysis, Function analysis, Pathway analysis, GO, KEGG, DO, Non-model species

## Abstract

**Background:**

Function genomic studies will generally result in lists of genes that may provide clues for exploring biological questions and discovering unanticipated functions, based on differential gene expression analysis, differential epigenomic analysis or co-expression network analysis. While tools have been developed to identify biological functions that are enriched in the genes sets, there remains a need for comprehensive tools that identify functional enrichment of genes for both model and non-model species from a different function classification perspective.

**Results:**

We developed AllEnricher, a tool that calculates gene set function enrichment, with user-defined updatable libraries backing up for both model and non-model species as well as providing comprehensive functional interpretation from multiple dimensions, including GO, KEGG, Reactome, DO and DisGeNET.

**Conclusions:**

AllEnricher incorporates up to date information from different public resources and provides a comprehensive resolution for biologists to make sense out of specific gene sets, making it an advanced open-source tool for gene set function analysis.

## Background

Functional genomics and large-scale genetic studies continuously generate a large number of gene sets (e.g. differentially expressed gene sets, co-expressed gene sets, or differential epigenomic modification gene sets, etc.). These gene sets are pivotal for elucidating molecular mechanisms in a biological system [1]. Investigating the relationship among these genes in the context of different function classification system provides clues for exploring biological questions and discovering unanticipated functions. Therefore, it is critical to characterizing gene-function relationships and mining gene-function associations of the gene sets.

Various kinds of databases have been developed for gene function classification. The most commonly used gene function database is the Gene Ontology (GO) [2]. Pathway-based database, like Kyoto Encyclopedia of Genes and Genomes (KEGG) [3] and Reactome [4], provide gene function interpretation through the perspective of biological reactions. Other databases like disease-based databases, such as Disease Ontology (DO) [5], DISEASE [6] and DisGeNET [7] were designed for molecular studies in disease. All these databases together provide comprehensive gene-function interpretations for the biologists.

Nonetheless, several analytic approaches based on different gene-function databases have been developed to decipher the biological significance of specific gene sets. Although proposed as the first generation of methods, Over-Representation Analysis (ORA) approaches still remain a commonly used method in exploring the functions implication of gene sets [1]. Based on this algorithm, many enrichment tools have been published, including GO-TermFinder [8], GOstat [9], WEGO [10], FunSet [11] for GO enrichment, KOBAS [12], clusterProfilers [13] for GO and KEGG enrichment, and DOSE [14] for disease enrichment. Though these tools can automatically calculate and visualize the significantly enriched function categories, various gene function analysis based on different tools and platforms make it complicated and tedious for biologists to choose and use. Therefore, collaborative tools like GO-Elite [15], MSigDB [16] and Enrichr [17] were developed to resolve these limitations. However, these tools either merely provide analysis for finite model species or the library they relied on are vulnerable to be out of date since their update depends on the timely maintenance of the author.

In this study, we developed a user-defined updatable application, which could be easily integrated into pipelines of functional genomic studies (RNA-seq, ATAC, BS-seq, etc.) and also can be incorporated into the five optimized public gene-function annotation collections (GO, KEGG, Reactome, DISEASE, and DisGeNET). Users of the application can update their local library to the latest version anytime they wish and decipher specific gene sets of both model and non-model species from appropriate gene function perspectives by enrichment analysis in just one single command.

## Implementation

### Public resources selection

**Fig. 1:**
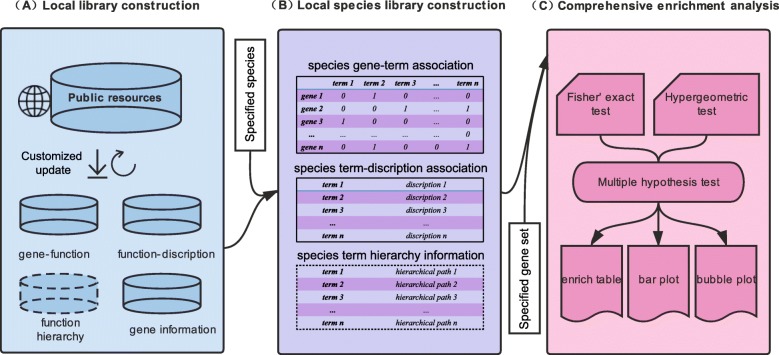
Design framework of AllEnricher software

**Fig. 2:**
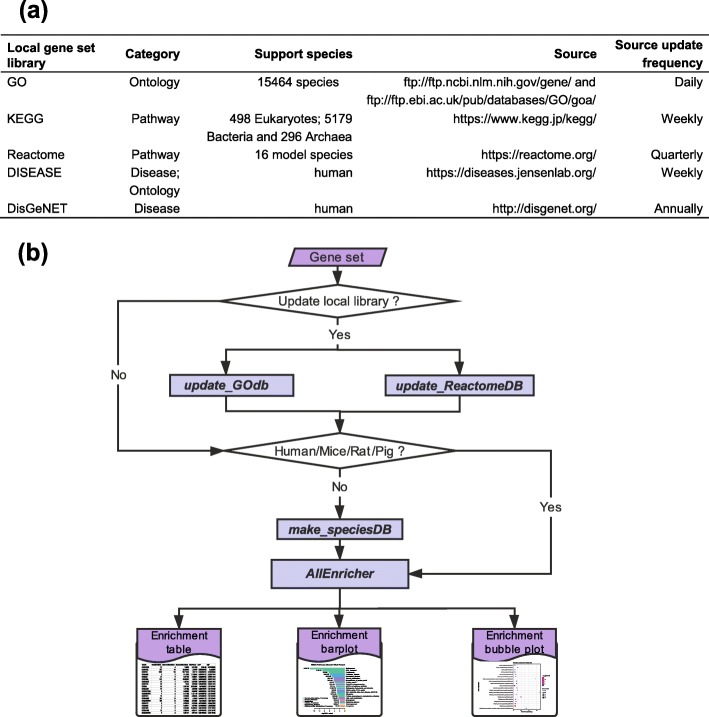
Overview of the AllEnricher software current version. **a** Details of the local gene set libraries **b** General workflow of *AllEnricher*

The design framework of this tool is shown in Fig. 1. To establish local libraries as back up for AllEnricher, we firstly selected a series of public resources. The optimized public database must be timely updated and should incorporate gene-function annotations for both model and non-model species. We finally integrated five public resources into the local library, including GO, KEGG, Reactome, DISEASE, and DisGeNET (Fig. 2a).

### Local gene ontology (GO) library construction

To obtain the GO annotation information of multiple species, we downloaded the public resource from NCBI and Gene Ontology Annotation (GOA). NCBI FTP (ftp://ftp.ncbi.nlm.nih.gov/) supplied the up to date GO annotation file, Gene Ontology (http://geneontology.org/) provided the obo file. The gaf file from GOA (https://www.ebi.ac.uk/GOA) provided all the GO annotations to proteins in the UniProt Knowledgebase (UniProtKB). The comprehensive gene information file supplied the corresponding relations between the NCBI official gene symbol and its gene ID (the unique identifier for a gene). All these files together make up the local GO library support for AllEnricher. We supplied a shell script to download and update this local database (*update_GOdb*) as per the user’s requirement. Libraries for specified species can be built based on the local established GO database by *makeDB.go.sh*.

### Local disease ontology (DO) library construction

The difficulty of DO analysis is to obtain the disease-gene annotation information. Though it has been developed as a standardized ontology for human disease, the Disease Ontology (http://disease-ontology.org/) falls short of up to date disease-gene annotation for the users. In 2013, The publication of Disease Gene Annotation (DGA, http://dga.nubic.northwestern.edu) [18] database provided an integrated environment to facilitate the analysis of disease–gene associations and explore potential gene interactions shared among multiple diseases. However, currently, it has been out of service. DISEASES database (https://diseases.jensenlab.org/) [6] is a weekly updated web resource that integrates evidence on human disease-gene associations from automatic text mining, manually curated literature, cancer mutation data, and genome-wide association studies. We, therefore, utilized the non-redundant gene-disease annotation from the text mining channel, knowledge channel and experiment channel and integrated it as the local DO resource. The update of the local DO database was integrated into the building of AllEnricher comprehensive database for human (*makeDB.do.v1.0.sh*).

### Local DisGeNET disease library construction

DisGeNET (http://disgenet.org/home/), which is another public gene-disease association database, is a discovery platform containing one of the largest publicly available collections of genes and variants associated with human diseases [7]. DisGeNET integrates data from expert curated repositories, GWAS catalogs, animal models and the scientific literature. We acquired all the gene-disease associations in DisGeNET and constructed the local DisGeNET disease library for AllEnricher. Local DISEASE and DisGeNET library only provide gene-disease annotations for humans, hence we merged and updated the progress into local library construction for human beings.

### Local KEGG pathway library construction

KEGG PATHWAY Database (https://www.kegg.jp/kegg/pathway.html) is a collection of manually drawn pathway maps representing current knowledge on the molecular interaction. It is a commercial subscription-based database that does not offer a pathway-gene annotation file for specified species, and for this reason, we wrote several R scripts to download pathway-gene associations for specified species. The functional hierarchies for specified species could also be obtained from KEGG BRITE Database. The establishment of the local KEGG library for specified species was integrated into the shell script *makeDB.kegg.v1.0.sh**.*

### Local Reactome library construction

Reactome (https://reactome.org/) is a free, open-source and open-data pathway database which provides comprehensive pathway knowledge to the biologist. We provide a script *update_ReactomeDB* to build and update the local Reactome database. First, we downloaded the pathway-gene annotation file of all the 16 kinds of species and then we filtered genes out of gene information files from NCBI. The local Reactome database for specified species was constructed based on these local resources by *makeDB.reactome.v1.0.sh*.

All the five gene-function libraries support for a specific species was built by script *make_speciesDB*. The accepted format for specifying genes is an official gene symbol from NCBI.

### Gene set enrichment analysis and visualization

Fisher’s exact test or hypergeometric test was employed to calculate the enrichment of the customized genes in the input gene set. The default genomic background gene sets for enrichment analysis were obtained from NCBI gene information. The False Discovery Rate (FDR) was controlled by multiple hypothesis testing with an alternative method of BH and FDR. The visualization of enrichment was accomplished via bar plots and bubble plots by R scripts. All the enrichment analysis and visualization steps had been integrated into the main program *AllEnricher*. The general workflow of AllEnricher is described in Fig. 2b.

## Results

### Command line application based on Unix

Shell, perl, and R were utilized to develop an easy to use command line application based on Unix environment to calculate gene enrichment of a user-provided gene set. Actually, the primary version of AllEnricher had been applied to several in house researches engaged in enrichment analysis of epigenetically modified gene sets and differentially expressed gene (DEG) sets of model species [19–21]. Here we used another two gene sets from previous studies to test AllEnricher.

### Case study 1: function enrichment analysis in investigations of human disease

The first example is a DEG set based on RNA-Seq generated from ten matched pairs of cancer and non-cancerous tissues from Hepatocellular Carcinoma (HCC) patients [22]. AllEnricher (Fisher’s Exact test, q-value < 0.05 by BH method) run on the 1378 DEGs identified in our study proved enrichment for cancer and tumorigenesis genes in both GO function and KEGG pathway analyses (Additional files 1 and 2), which are consistent with the main findings in this study. Moreover, DisGeNET disease enrichment analyses also reveal significant enrichment of genes associated with several kinds of carcinomas, with liver carcinoma emerging as the most enriched disease. These results validate the reliabiliy of AllEnricher and highlight specifically expressed genes in HCC that may confer important clues for understanding the molecular mechanisms of HCC pathogenesis.

### Case study 2: function enrichment analysis in studies refers to non-model species

To illustrate how AllEnricher performs in gene set enrichment analysis on non-model species, we analyzed the RNA-seq data of golden snub-nosed monkeys (*Rhinopithecus roxellana*) living in the wild during both the winter and summer season [23]. We identified 2967 genes differentially expressed in different seasons using a well-established protocol [24]. To decipher the functional implications of this DEG list, *make_sepciesDB was used* to construct the local KEGG library for this species. As a result, 8327 genes annotated to 329 pathways constituted the unique local KEGG pathway library for the golden snub-nosed monkeys. KEGG pathway enrichment analysis by AllEnricher was applied to the seasonal DEGs (Hypergeometric test, q-value < 0.05 by BH method). Intriguingly, a wide range of associated physiological and metabolic pathways was enriched, including thermogenesis, oxidative phosphorylation, and pentose phosphate pathway (Additional files 3 and 4). The results obtained for seasonal stress and corresponding physiological response of golden snub-nosed monkeys in winter, provides a reasonable mechanism for the explaination of the adaption to the cold winter when food was scarce in the wild.

### Comparison to other similar state-of-the-art tools

In order to investigate the similarities that exist between the AllEnricher and other similar tools, AllEnricher was compared to Enrichr, GO-Elite, clusterProfilers, and FunSet, which are the four leading tools that provide gene set enrichment analysis from comprehensive perspectives. Below is a summary of some important features of all the four tools, which refer to support species and libraries, library update, availability, ie. pipeline embeddable or customized background list (Table 1).

**Table 1:** Comparison to other similar state-of-the-art tools

Name	Support species	Support Libraries	Library update	Availability	Pipeline embeddable	Customized background list
AllEnricher	Model species and non-model species	GO, KEGG, Reactome, DISEASE, and DisGeNET	Customized	Standalone (https://github.com/zd105/AllEnricher)	Yes	Yes
Enrichr	Human, mouse and rat	GO, KEGG, GEO, InterPro, WikiPathways, MGI, Chromosome Location, Genome Browser PWMs, TargetScan, Reactome, BioCarta, TRANSFAC and JASPAR PWMs, Epigenomics Roadmap, ENCODE, ChEA, PPI databases, NURSA, CORUM, LINCS L1000, DEPOD, HumanCyc, NCI-Nature, Panther, KEA, HPO, GeneSigDB, CMAP, OMIM, VirusMINT, Achilles, dbGaP, Human Gene Atlas, Mouse Gene Atlas, ESCAPE, GTEx, HMDB and HomoloGene	Developer dependent	Web (http://amp.pharm.mssm.edu/Enrichr/)	No	No
GO-Elite	Over 60 species	GO, KEGG, GEO, InterPro, WikiPathways, MGI, Disease Ontology, GOSlim, Amadeus Metazoan compendium, PAZAR and AltAnalyze	Developer dependent	Standalone, Web (http://www.genmapp.org/go_elite/)	Yes	No
clusterProfilers	19 species	GO and KEGG	Developer dependent	Standalone (https://bioconductor.org/packages/release/bioc/html/clusterProfiler.html)	Yes	No
FunSet	11 species	GO	Developer dependent	Standalone, Web(http://funset.uno/)	Yes	Yes

(1) Comprehensive function interpretation support: The same gene set provided by users could be interpreted from multiple aspects according to their purpose, which including Gene Ontology, KEGG pathway, Reactome pathway, Disease Ontology, and DisGeNET disease. Although the coverage of library collections is less than Enrichr and GO-Elite, gene-function annotations based on various kinds of database are planned to integrate as local libraries of AllEnricher to satisfy requirements of researches in a different field in the future, based on current program framework.

(2) Model species and non-model species support: The 1.0 version of AllEnricher already provides established local libraries for four kinds of most commonly studied model species, including humans (*Homo sapiens*), mouse (*Mus musculus*), Rat (*Rattus norvegicus*) and Pig (*Sus scrofa*). The number of species that supports the application is largely dependent on the public resources which the local library of AllEnricher is based on. Users could extend the supports for specific species by constructing the corresponding local species libraries. AllEnricher current version supports 15,464 species for GO enrichment, 5973 species for KEGG enrichment and merely human for disease enrichment (Fig. 1a), far more species than the other four tools. Supporting the analysis of various non-model species is the typical feature of AllEnricher.

(3) Customized library updates: The local library of AllEnricher was built based on frequently updated public resources (Fig. 2a). Compared to the other four similar tools, which library updates depend on the developers, several simple commands for customized library updates were designed. Therefore, users could obtain the latest data as they need.

(4) Customized background gene list: Enrichment analysis requires a background gene list. In general, researchers would take all the genes from the genome of specific species as the background gene list. However, the background gene list should be merely part of the genes from the whole genome in some cases. For example, when a DEG set is generated from samples of a specified tissue, where some parts of genes never expressed due to the high differentiation of cells, they should be excluded from the background gene list of enrichment analysis. AllEnricher provides flexible solutions to satisfy the application scenarios of the user-defined background gene list.

## Conclusions

This study has demonstrated that a command line application based on the general Unix environment provides a robust way to carry out gene function enrichment, with support for multiple species and comprehensive functional perspectives. AllEnricher incorporates up to date information from different public resources and provides a comprehensive tool for biologists to make sense of specific gene lists. In summary, the wide application scenarios of AllEnricher makes it an advanced tool for gene set function enrichment analysis.

## Supplementary information

**Additional file 1.** Result tables enrichment analysis in case study 1.

**Additional file 2.** Result figures enrichment analysis in case study 1.

**Additional file 3.** Result figures enrichment analysis in case study 2.

**Additional file 4.** Result figures enrichment analysis in case study 2.

## Data Availability

AllEnricher source code is freely available in the GitHub repository (https://github.com/zd105/AllEnricher). The computing code is licensed under MIT. The online readme is the most extensive and continually updated source of documentation for AllEnricher, covering installation and user guide.

## Abbreviations

DEG: Differentially expressed gene
DO: Disease Ontology
FDR: False Discovery Rate
GO: Gene Ontology
HCC: Hepatocellular Carcinoma
KEGG: Kyoto Encyclopedia of Genes and Genomes
ORA: Over-Representation Analysis
