# Supplementary figures and images for: AllEnricher: a comprehensive gene set function enrichment tool for both model and non-model species
Source: BMC Bioinformatics. 2020 Mar 17;21:106. doi: 10.1186/s12859-020-3408-y (PMC7076970; doi:10.1186/s12859-020-3408-y)

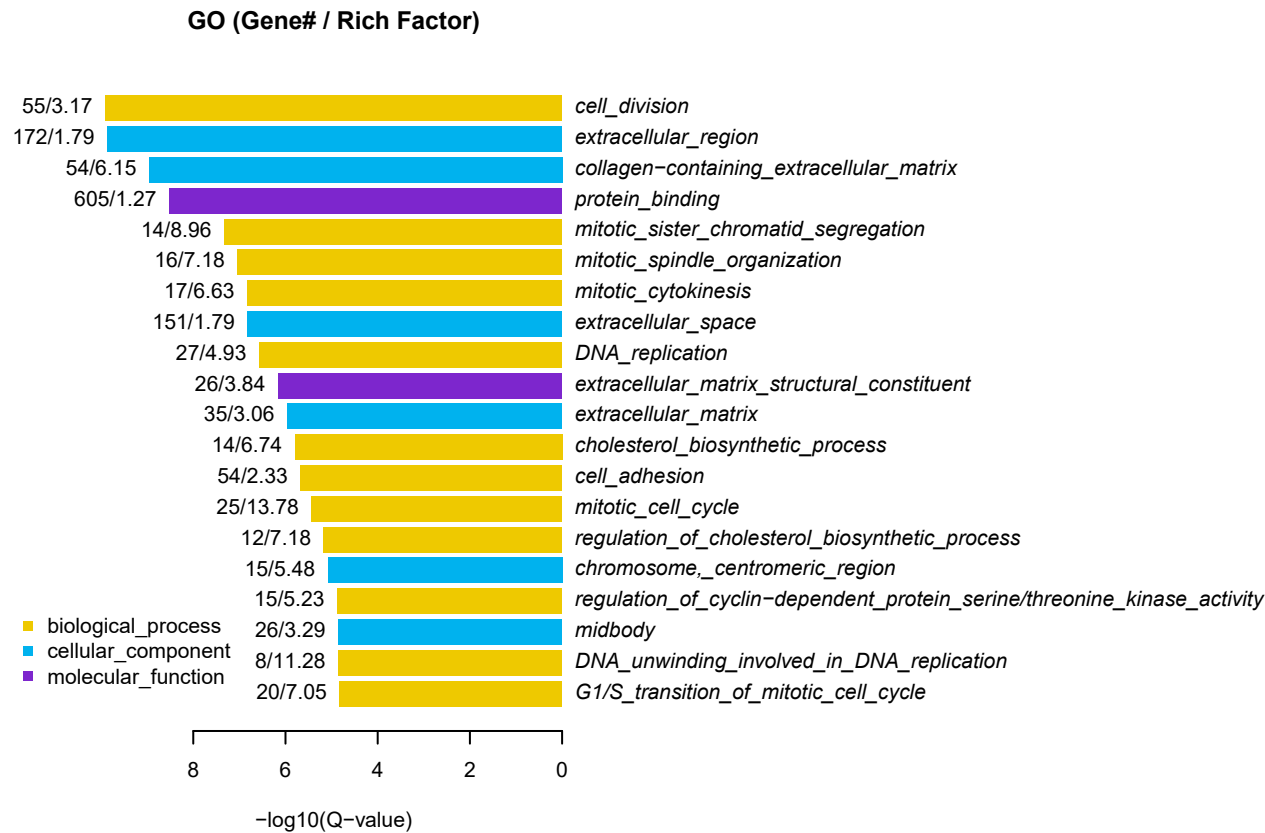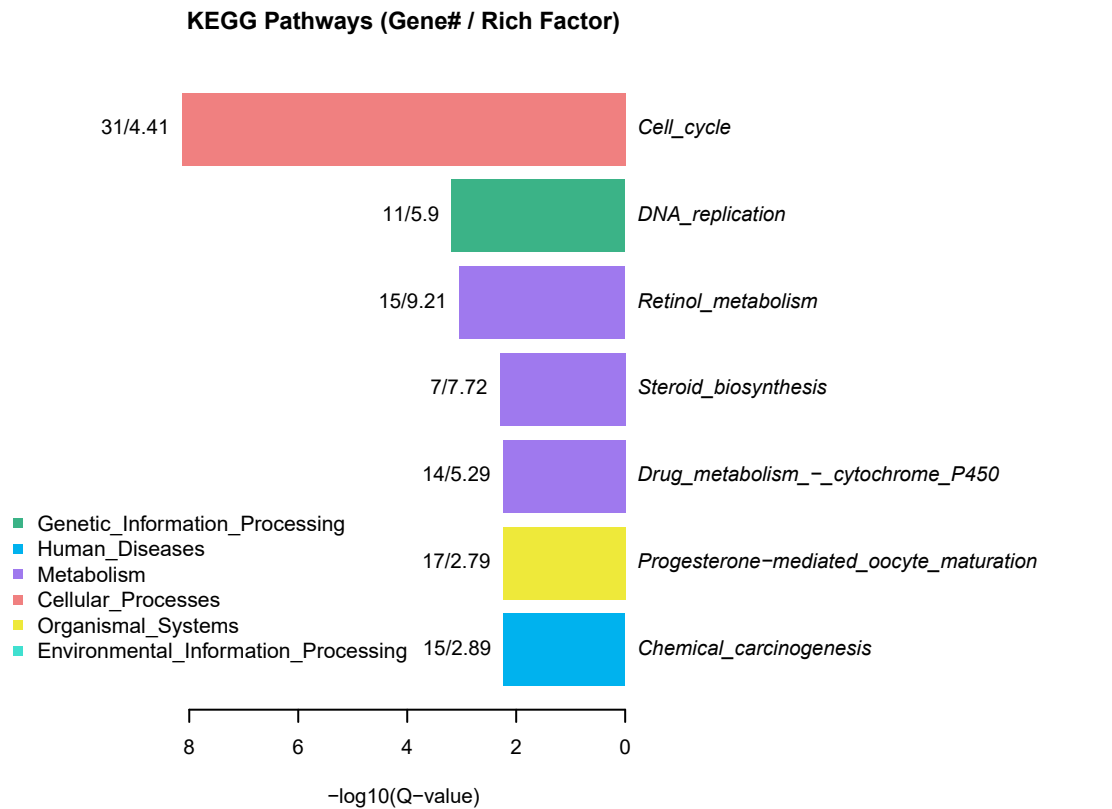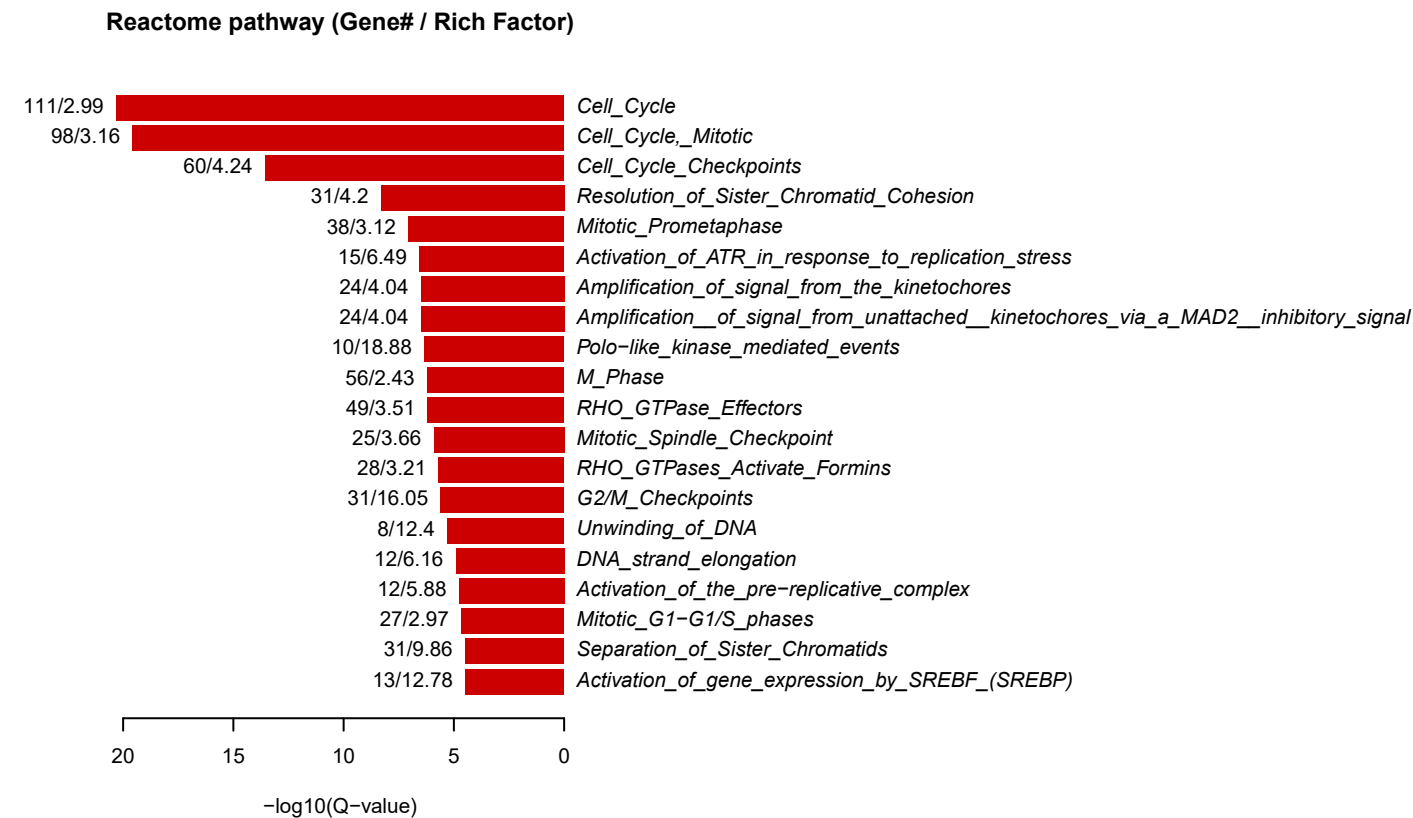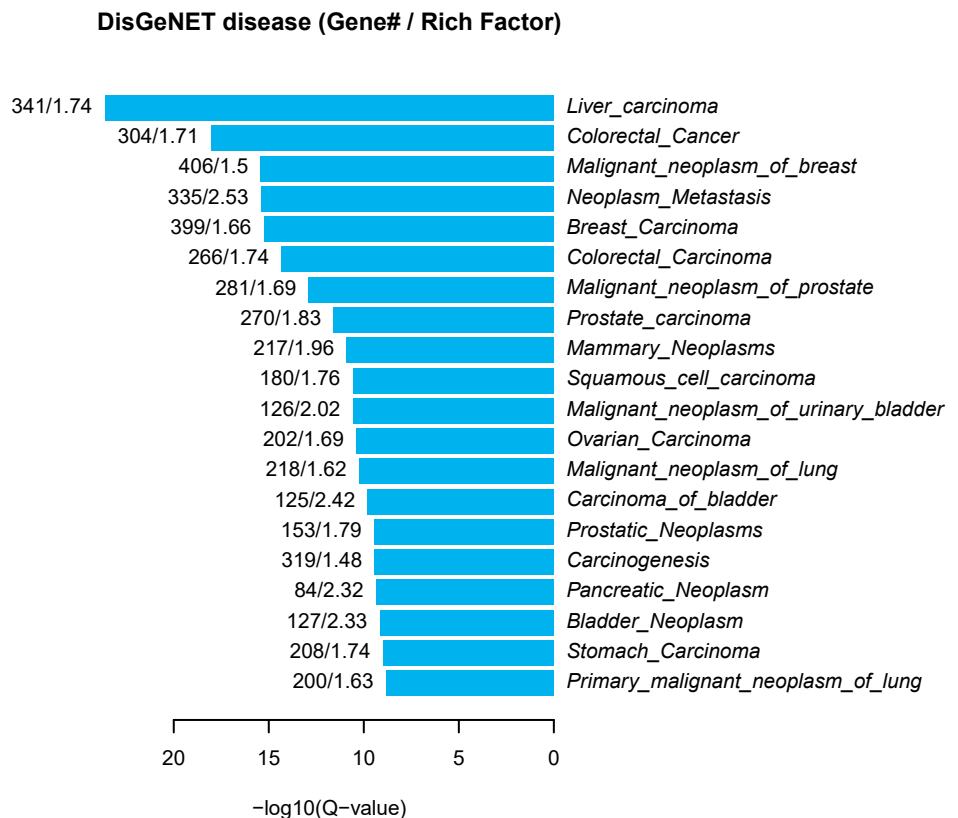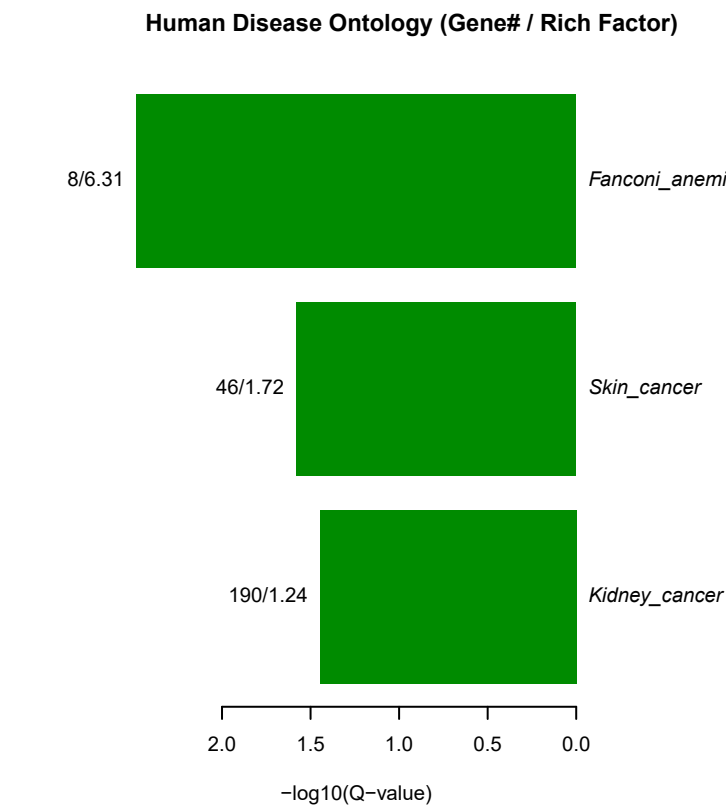

Supplement: Supplementary file 2 — Additional file 2. Result figures enrichment analysis in case study 1. [file 12859_2020_3408_MOESM2_ESM.pdf]

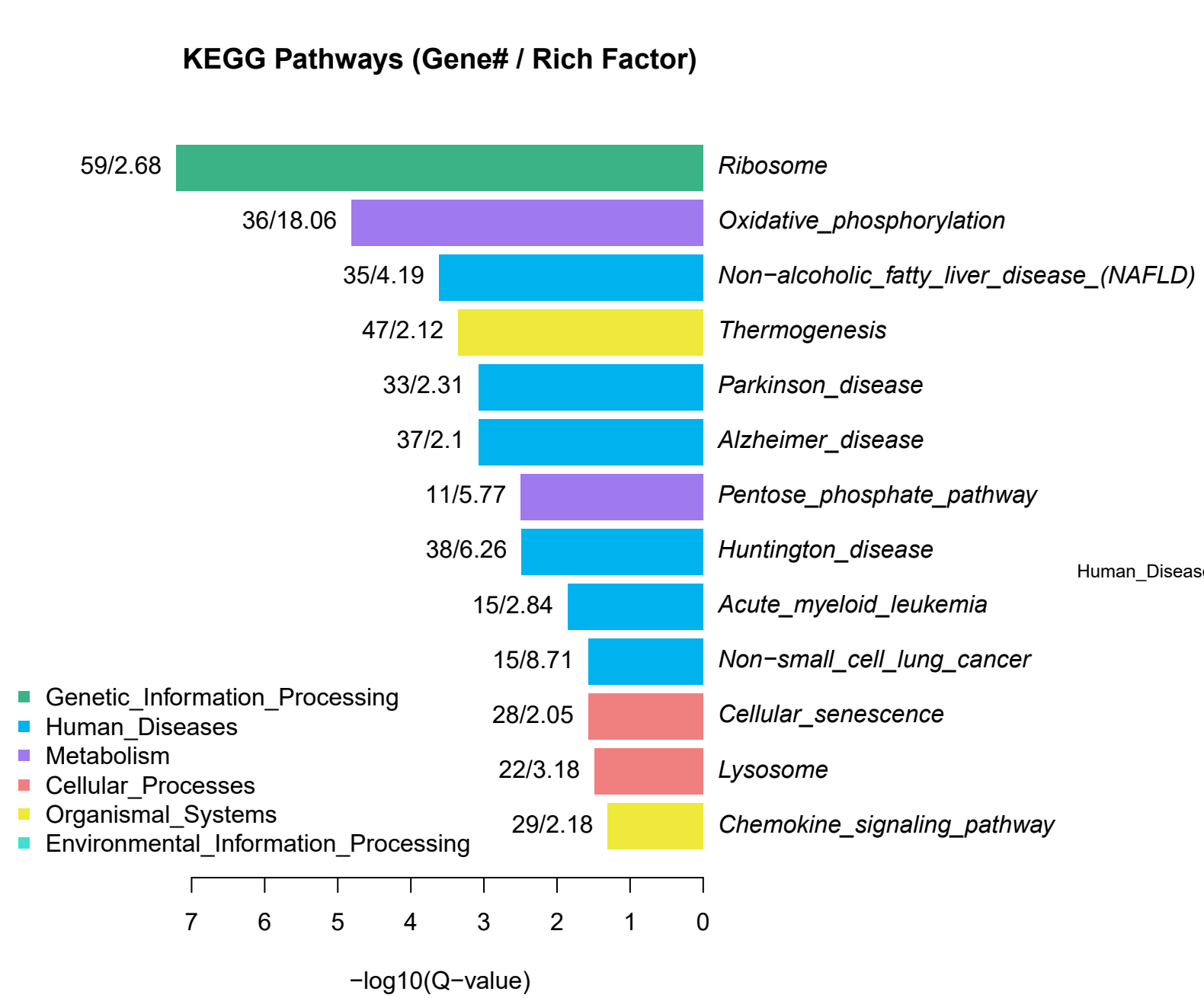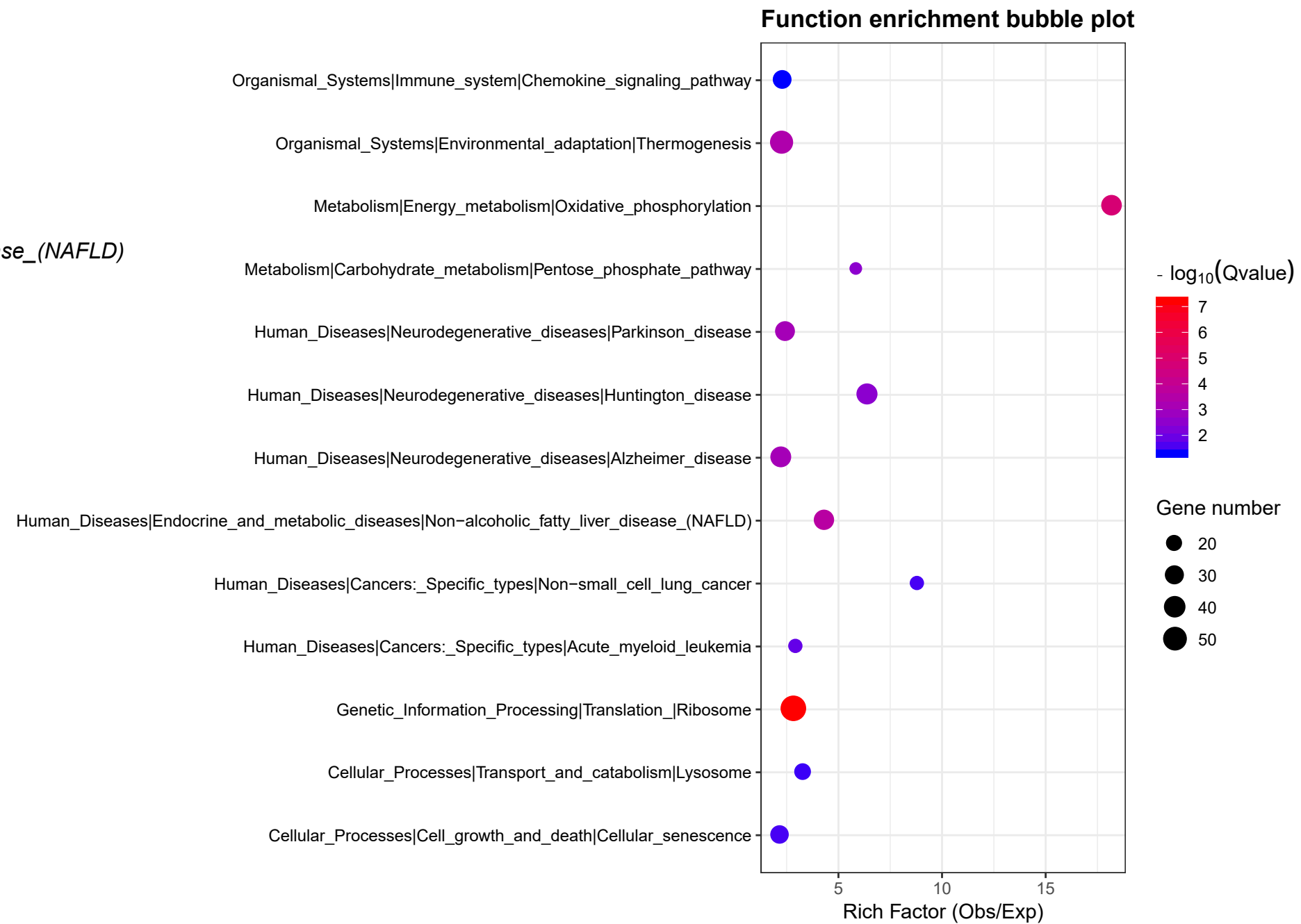

Supplement: Supplementary file 4 — Additional file 4. Result figures enrichment analysis in case study 2. [file 12859_2020_3408_MOESM4_ESM.pdf]
